# Identification of key genes as predictive biomarkers for osteosarcoma metastasis using translational bioinformatics

**DOI:** 10.1186/s12935-021-02308-w

**Published:** 2021-12-02

**Authors:** Fu-peng Ding, Jia-yi Tian, Jing Wu, Dong-feng Han, Ding Zhao

**Affiliations:** 1 Department of Orthopedics Surgery, The First Hospital of Jilin University, Changchun, 130021 China; 2 Department of Emergency Medicine, The First Hospital of Jilin University, Changchun, 130021 China; 3 Department of Reproductive Medicine and Center for Prenatal Diagnosis, The First Hospital of Jilin University, Changchun, 130000 China; 4 Department of General Practice, The First Hospital of Jilin University, Changchun, 130000 China

**Keywords:** Osteosarcoma, Metastasis, ARHGAP25, TCGA, WGCNA

## Abstract

**Background:**

Osteosarcoma (OS) metastasis is the most common cause of cancer-related mortality, however, no sufficient clinical biomarkers have been identified. In this study, we identified five genes to help predict metastasis at diagnosis.

**Methods:**

We performed weighted gene co-expression network analysis (WGCNA) to identify the most relevant gene modules associated with OS metastasis. An important machine learning algorithm, the support vector machine (SVM), was employed to predict key genes for classifying the OS metastasis phenotype. Finally, we investigated the clinical significance of key genes and their enriched pathways.

**Results:**

Eighteen modules were identified in WGCNA, among which the pink, red, brown, blue, and turquoise modules demonstrated good preservation. In the five modules, the brown and red modules were highly correlated with OS metastasis. Genes in the two modules closely interacted in protein–protein interaction networks and were therefore chosen for further analysis. Genes in the two modules were primarily enriched in the biological processes associated with tumorigenesis and development. Furthermore, 65 differentially expressed genes were identified as common hub genes in both WGCNA and protein–protein interaction networks. SVM classifiers with the maximum area under the curve were based on 30 and 15 genes in the brown and red modules, respectively. The clinical significance of the 45 hub genes was analyzed. Of the 45 genes, 17 were found to be significantly correlated with survival time. Finally, 5/17 genes, including ADAP2 (P = 0.0094), LCP2 (P = 0.013), ARHGAP25 (P = 0.0049), CD53 (P = 0.016), and TLR7 (P = 0.04) were significantly correlated with the metastatic phenotype. In vitro verification, western blotting, wound healing analyses, transwell invasion assays, proliferation assays, and colony formation assays indicated that ARHGAP25 promoted OS cell migration, invasion, proliferation, and epithelial–mesenchymal transition.

**Conclusion:**

We identified five genes, namely ADAP2, LCP2, ARHGAP25, CD53, and TLR7, as candidate biomarkers for the prediction of OS metastasis; ARHGAP25 inhibits MG63 OS cell growth, migration, and invasion in vitro, indicating that ARHGAP25 can serve as a promising specific and prognostic biomarker for OS metastasis.

**Supplementary Information:**

The online version contains supplementary material available at 10.1186/s12935-021-02308-w.

## Background

Osteosarcoma (OS) is a prevalent malignant and highly invasive cancer that is primarily observed in children and adolescents [1]. OS metastasis is the most common cause of cancer-related mortality. Despite advances in combined therapy (surgery, radiotherapy, systemic multi-agent chemotherapy, and immunotherapy), the 5-year survival rate of OS patients with localized tumors remains at 60–70%, while that for metastatic and recurrent patients is < 20% [2, 3]. Several molecular mechanisms have been identified to play a role in the OS metastasis cascade, such as the Wnt/β-catenin pathway [4, 5], PI3K/Akt/mTOR [6] and Notch signaling [7]. Many genes have been identified as potential biomarkers for the prediction and treatment of OS metastasis [8–10]. However, the mechanism underlying OS metastasis remains unclear. Thus, a better understanding of the mechanism of OS metastasis is urgently required to identify more effective and specific biomarkers for early prediction, survival assessment, and treatment.

Data-driven approaches, such as gene microarrays, have been employed to identify the driver genes of OS genesis and metastasis [11, 12]. Several studies screened genes based on their expression patterns and analyzed their function using Gene Ontology (GO) or Kyoto Encyclopedia of Genes and Genomes (KEGG). However, with this method, a number of potential interconnections among genes are missed. Weighted gene co-expression network analysis (WGCNA) is a systematic biology method to cluster highly correlated genes into one module and to relate the module to clinical traits. Therefore, WGCNA is more beneficial for identifying driver genes in prognosis and therapy [13].

The support vector machine (SVM) classifier is a specific method of machine-learning. It has been widely applied in selecting biomarker genes for disease classification and prediction because of its high accuracy and the ability to identify the multivariate statistical properties of data between two different groups [14]. Receiver operating characteristic curve analysis was used to evaluate the performance of the SVM classifier. It is widely used as a valid statistical method to determine the clinical utility of biomarkers [15].

In this study, we aimed to provide a bioinformatics method to identify the most relevant genes as potential biomarkers in OS metastasis and to employ biological methods to verify its effectiveness.

## Materials

### Data information

We obtained the gene expression profiles of GSE33382 and GSE21257 from the NCBI Gene Expression Omnibus (GEO; https://www.ncbi.nlm.nih.gov/geo/). GSE33382 and GSE21257 consist of 84 and 53 OS samples, respectively, based on the GPL10295 Illumina Human-6 v2.0 Expression BeadChip Platform (Illumina Inc., CA, USA). The Cancer Genome Atlas (TCGA) and TARGET-OS (Children’s Oncology Group and the Hospital for Sick Children in Toronto, Canada) data matrix (https://ocg.cancer.gov/programs/target/data-matrix) were used to validate the SVM classifier. The R packages illuminaio and lumi were used to process and analyze the raw data.

### In silico analysis

WGCNA was performed on the genes that appeared in TCGA and GEO, and GO and KEGG enrichment analyses were conducted to explore the biological relevance of the key modules in WGCNA. CytoHubba on Cytoscape v3.6.0 (http://www.cytoscape.org/) was used to construct a protein–protein interaction (PPI) analysis of key WGCNA modules. An SVM classifier was employed for the prediction and evaluation of key genes involved in OS metastasis. The relationship between the key genes and OS prognosis was analyzed using Kaplan–Meier survival curve analysis. To evaluate the prognostic effect of the key genes in OS patients, data from TCGA were used to verify their expression levels in OS.

### Modules identification and preservation analysis

As a training set, the raw data of GSE33382 and GSE21257 were used to construct co-expression networks and to screen hub genes. Distance in Pearson’s correlation matrices and average linkage between different samples were used to cluster samples and assess the microarray quality. As a result, three samples (GSM825681, GSM531298, and GSM825697) were excluded in subsequent analyses (Additional file 3: Fig. S1). The radiometric multiresolution analysis algorithm was used for background correction. To evaluate the impact of power value on the mean connectivity and scale independence, the function “softConnectivity” in WGCNA package was used, and the “randomly selected genes” parameter was set at 16,000. The “pick SoftThreshold” function of WGCNA was used to evaluate the best soft thresholding power for constructing networks. Then, we calculated the dissimilarity of module eigengenes to provide a cutline for module merging.

The stability of the identified modules was tested using fragments per million expression data of 86 samples from TCGA dataset. We conducted preservation analysis using 9081 common genes in both the training and test datasets with the “nPermutations” parameter set at 200.

### Identification of key modules and functional annotations

We correlated module eigengenes with clinical traits to identify the relevant modules. In the present study, clinical traits refer to metastatic conditions. A linear regression model was used to evaluate the correlation between gene expression and clinical traits. We performed the functional enrichment analysis of key modules using “clusterProfile” package in R.

### Identification of hub genes in key modules

In the present study, the relationship between module connectivity and metastasis traits was evaluated to identify hub genes in the key modules. We also constructed a PPI network of genes in key modules. CytoHubba, a Cytoscape plugin app, sorts the genes by analyzing 12 parameters, namely DEGREE, EcCentricity, MCC, RADIALITY, STRESS, CLOSENESS, DMNC, MNC, BETWEENNESS, EPC, BOTTLENECK, and ClusteringCoefficient. The top 50 genes, ranked by each parameter, were recorded. We explored the genes in the top 50 sorting by 8 or more parameters to identify the essential hub genes in the functional network as the hub genes with more essential in the functional network [16]. Furthermore, we screened the differentially expressed genes (DEGs) using the “limma” R package. The common hub genes identified in the co-expression network, PPI network, and DEGs were considered as key genes for further analysis and validation [17, 18].

### Prediction and evaluation of key genes for OS metastasis by SVM classifier

The samples GSE33382 and GSE21257 were ranked randomly, and 75% of the samples were selected to train the SVM classifier. In each key module, an increment of five genes was added to the classifier to separate metastatic OS from non-metastatic OS. The remaining 25% of samples were used as validation sets. Sensitivity, specificity, area under the curve (AUC), positive predictive value, and the negative predictive value were calculated to evaluate the SVM classifier.

### Survival analysis and efficacy evaluation

TCGA database was used to perform the survival analyses. We performed survival and relapse-free survival analyses for all key genes from the brown and red modules using the survival package in R. The relationship between the key genes and OS prognosis was analyzed using Kaplan‑Meier survival curve analysis. Genes with a value of P < 0.05 were considered to be statistically significant and used for further validation.

### Cell lines

The OS cell lines MG-63 (BNCC338584) and U2OS(BNCC352039) used here were purchased from Beina Cell Bank (Beijing, China). Cells were cultured in Dulbecco’s Modified Eagle Medium (HyClone, UT, USA) containing 10% fetal bovine serum (FBS; Gibco, TX, USA) at 37 °C and 5% CO_2_. pcDNA-ARHGAP25 plasmids were designed and synthesized by Sangon Biotech (Shanghai, China).

### Cell transfection

MG63 and U2OS cells were seeded at a density of 3 × 10^5^ cells/well in 6-well plates. For ARHGAP25 overexpression, the cells were transfected with an expression vector containing the ARHGAP25 coding sequence or pcDNA3.1 vector control (NC; 1.5 μg/well). Transient transfection was performed using Lipofectamine 2000 (11668030, Invitrogen, CA, USA).

### Quantitative real-time polymerase chain reaction

Real-time polymerase chain reaction (RT-PCR) was performed to quantitatively identify ARHGAP25 expression levels. Briefly, total RNA was extracted, and cDNA was synthesized from RNA by reverse transcription, quantitative real-time PCR was undertaken using qPCR SYBR Green Master Mix for (11201ES03, Yeasen, China). The primer sequences used for RT-qPCR were 5ʹ-CCTGGAGCACGGCCGGAATG-3ʹ (sense) and 5ʹ-ACCACGGGCTCTGGGAGGTC-3ʹ (antisense) for ARHGAP25, and 5ʹ-ACAACTTTGGTATCGTGGAAGG-3ʹ (sense) and 5ʹ-GCCATCACGCCACAGTTTC-3ʹ (antisense) for GAPDH.

### Western blot analysis

Cells were lysed using RIPA buffer (Beyotime Institute of Biotechnology, Jiangsu, China) with protease inhibitors. The proteins were separated by SDS-PAGE and transferred to polyvinylidene difluoride (PVDF) membranes (0.45 µm, IPFL00010, Millipore Corporation, USA), which were blocked with non-fat milk and probed with primary antibodies, followed by horseradish peroxidase-conjugated IgG. Protein signals were visualized using an enhanced chemiluminescence detection kit (DW101; TransGen Biotech, Beijing, China). GAPDH was used as a reference protein. Primary antibodies against TWIST1 (25465), anti-E-cadherin (20874), anti-vimentin (10366-1), and GAPDH (60004-1) were purchased from Proteintech (NJ, USA).

### Cell viability assay

Transfected MG63 or U2OS cells were seeded in 96-well plates at a density of 0.3 × 10^3^ cells/well and cultured at 37 °C and 5% CO_2_ for 24, 48, and 72 h with diluted Cell Counting Kit 8 (CCK8; 1:10). One hundred microliters of diluted CCK8 was added to each well at each time point. Absorbance was detected using a Fluostar Omega microplate reader (BMG Labtech, Ortenberg, Germany) at a wavelength of 450 nm.

### Wound healing assay

Transfected cells were seeded in 6-well plates at a density of 8 × 10^5^ cells/well and cultured until they reached 90% confluence. A sterile 200 μL tip was used to create a gap in the cells. Each well was washed with phosphate-buffered saline three times and further cultured in serum-free medium for 24 h. Finally, gap size was photographed and measured with a microscope (Olympus, Tokyo, Japan).

### Colony formation assay

Transfected cells were trypsinized, and 0.5 × 10^3^ cells were plated in 24-well plates and incubated at 37 °C for 7 days. A dyeing solution containing 0.1% crystal violet and 20% methanol was used. Colonies were counted and analysed using the ImageJ software v1.52a (http://rsb.info.nih.gov/ij/).

### Transwell assays

Cell invasion was assessed using 24-well plates with transwell chambers. The upper chambers were coated with Matrigel (dilution 1:2; BD Biosciences, NJ, USA) and incubated for 1 h at 37 °C before cell cultures. Cells (5 × 10^4^) in serum-free medium were plated in the upper chambers. The lower chambers were filled with complete medium containing 10% FBS. Following 24 h of incubation, invasive cells in the lower chamber were washed, fixed, and stained with 0.1% crystal violet. Invasive cells were counted under a microscope (Olympus).

### Statistical analysis

All results are expressed as the mean ± standard deviation. Student’s t-test was used to compare groups. Statistical significance: *P < 0.05, **P < 0.005, and ***P < 0.001. Analyses were performed using the GraphPad Prism software v8.0.

## Results

### Weighted co-expression network construction and module preservation analysis

**Fig. 1:**
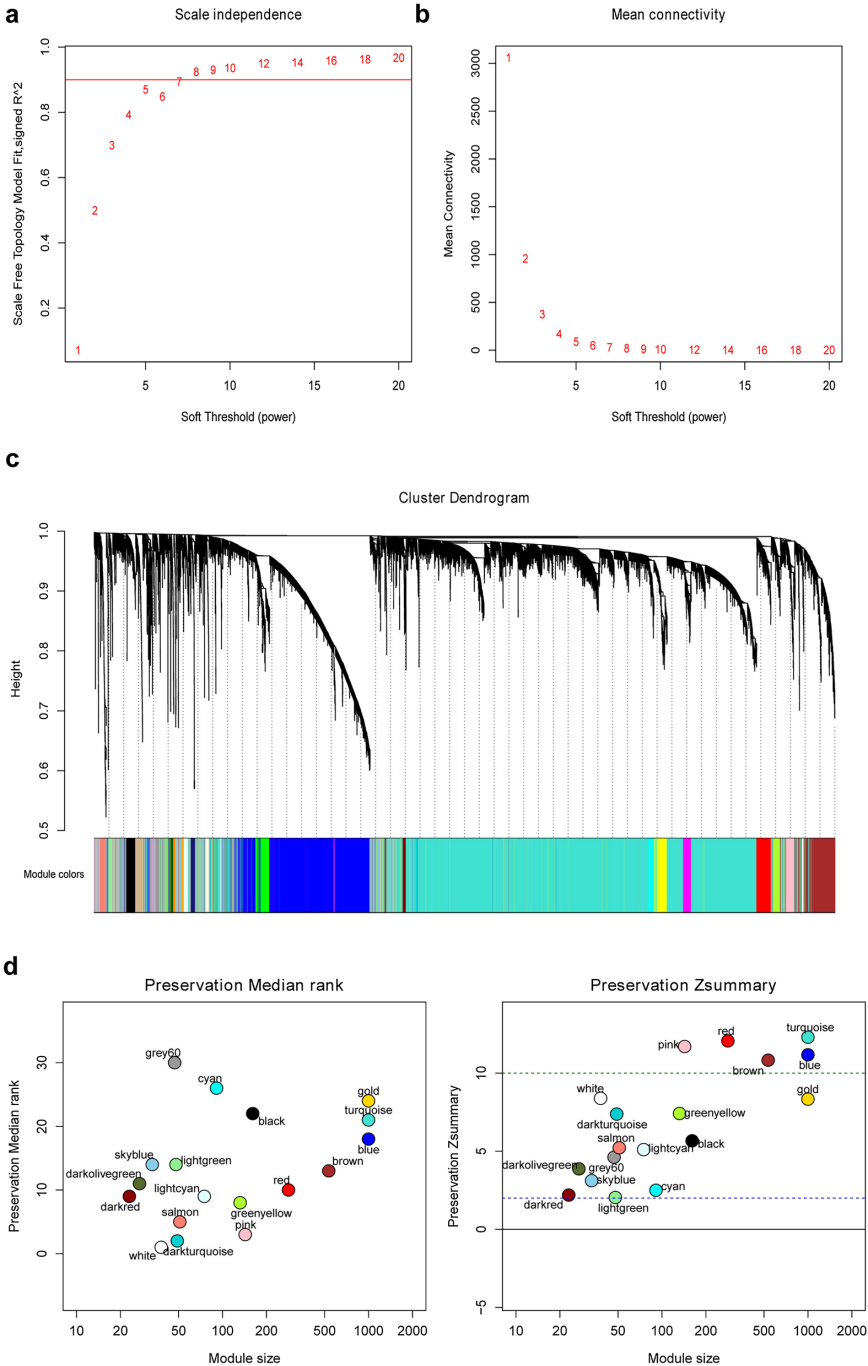
Co-expression modules construction and module preservation analysis. **a** Analysis of scale-free fit index for various soft-thresholding powers. **b** Analysis of mean connectivity for various soft-thresholding powers. **c** Color coding of co-expression network modules for mRNAs. **d** MedianRank (left) and Zsummary (right) statistics of the most variant gene modules in module preservation

We performed WGCNA on 15,040 genes after removing three outlier samples (GSM825681, GSM531298, and GSM825697; Additional file 3: Fig. S1), with the most appropriate soft threshold power of 5 (R2 = 0.87; Fig. 1a, b). Finally, 18 co-expression modules were identified (Fig. 1c).

By using summary preservation statistics, we evaluated whether the co-expression modules were stable from the training dataset (GSE33382 and GSE21257) to TCGA test dataset. Thirteen modules with a Zsummary statistic < 10 were defined as poor preservation. The pink, red, turquoise, blue, and brown modules were consistently stable and were selected for further analysis (Fig. 1d).

### Identification of key modules and functional annotation

**Fig. 2:**
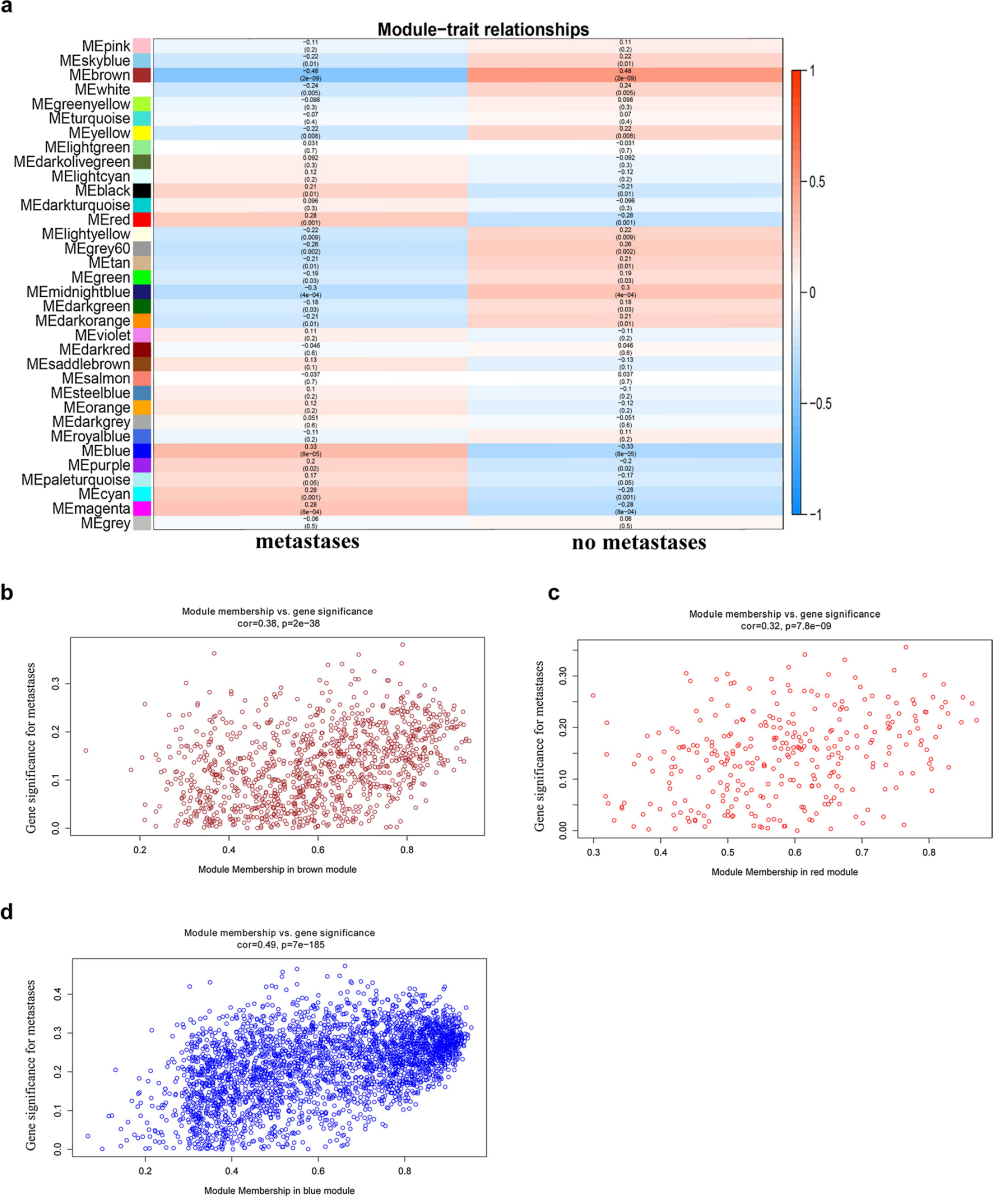
Heat map of correlation between eigengene modules and OS metastasis. **a** Three modules (brown, blue and red) had significant correlation with OS metastasis (P < 0.05). Scatter plot of eigengene modules in the brown (**b**), red (**c**) and blue (**d**) modules

To identify the most significant modules, we analyzed the relevance between each module and OS metastasis. In the five preserved modules, three modules (brown, blue, and red) were significantly correlated with OS metastasis (Fig. 2). Therefore, the genes contained in the brown, blue, and red modules were further analyzed.

**Fig. 3:**
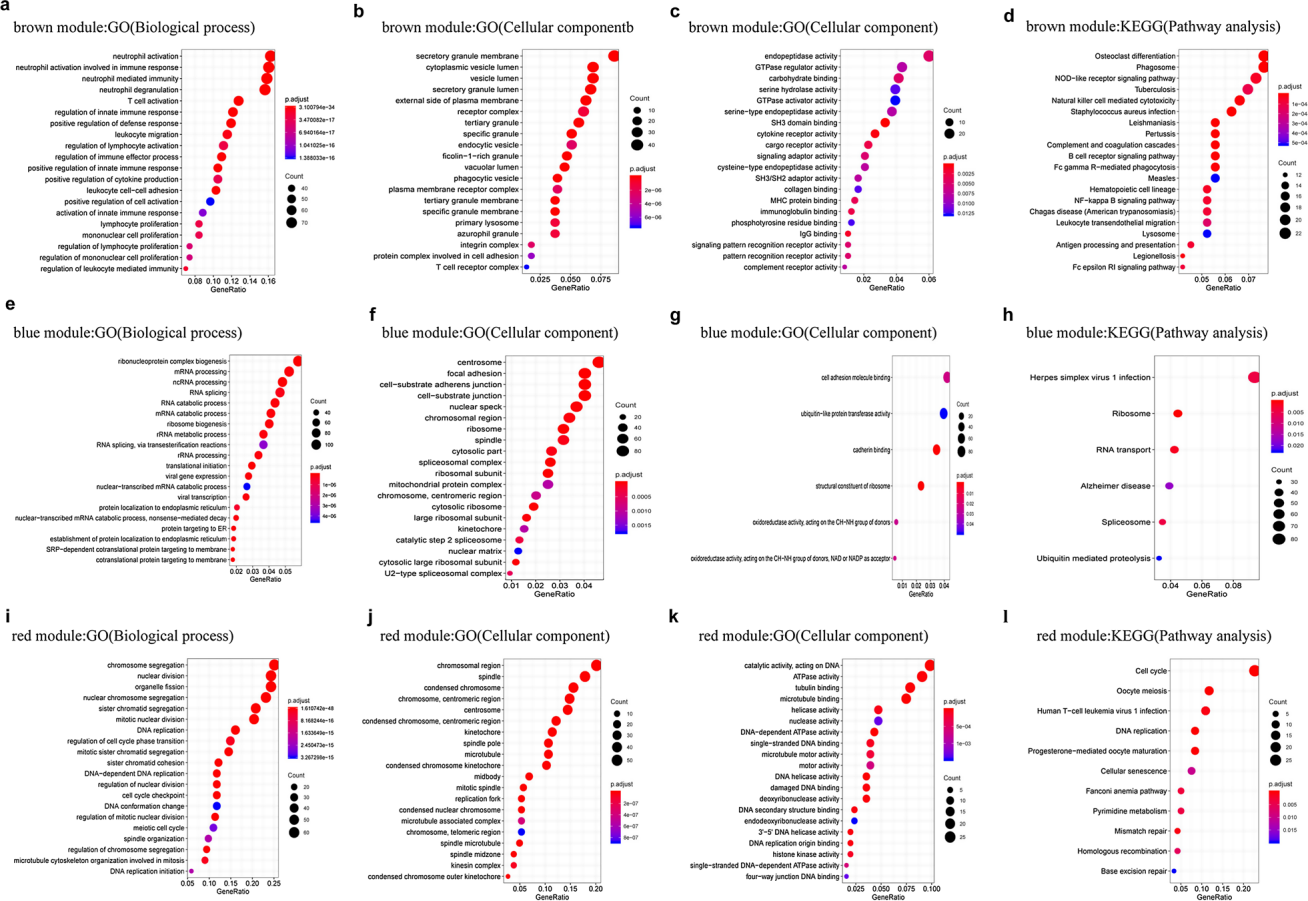
GO functional and significant KEGG pathways enrichment in the key modules. **a**–**c** GO functional enrichment of genes in the brown module. **d** Significant KEGG pathways enrichment in the brown module. **e**–**g** GO functional enrichment of genes in the blue module. **h** Significant KEGG pathways enrichment in the blue module. **i**–**k** GO functional enrichment of genes in the red module. **l** Significant KEGG pathways enrichment in the red module

Furthermore, GO and KEGG enrichment analyses were conducted to explore the biological relevance of the three modules. The results showed that genes in the brown module were primarily enriched in immunity, regulation of osteoclast differentiation, NOD-like receptor signaling pathway, and NF-κB pathway (Fig. 3a–d). Genes in the blue module were predominantly enriched in the regulation of RNA processing and cell adhesion molecular binding (Fig. 3e–h). Genes in the red module were mainly enriched in the regulation of DNA-associated activity and cell cycle (Fig. 3i–l).

### Identification of genes in key modules

**Fig. 4:**
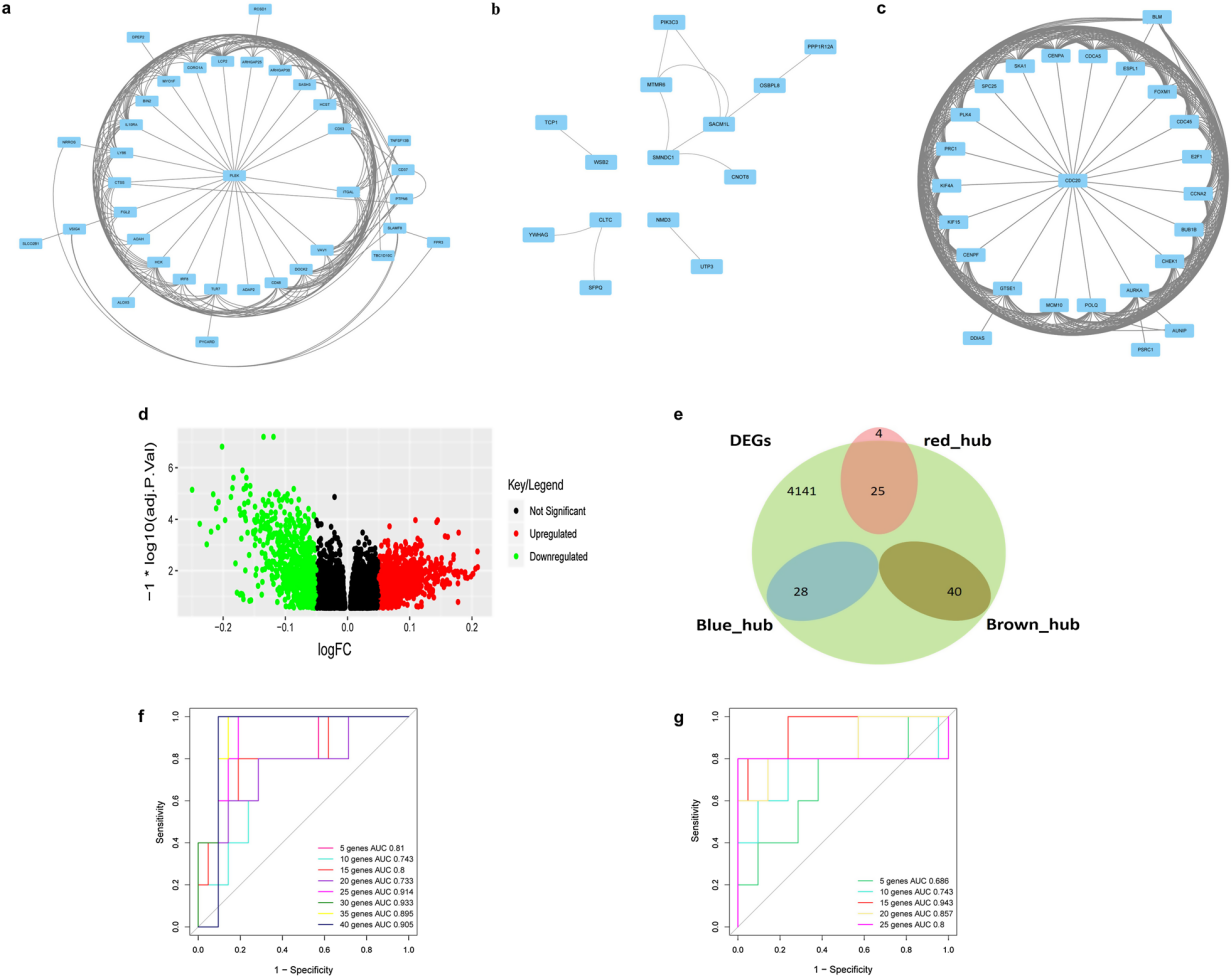
The identification of key genes in key modules. PPI network of the genes in brown (**a**), blue (**b**), and red (**c**) modules. **d** Volcano plot visualizing DEGs between metastasis and non-metastasis OS samples. **e** Identification of common genes between DEGs and the key modules by overlapping them. Receiver operating characteristic curve of support vector machine classification and showed the diagnostic efficiency of genes in brown (**f**) and red (**g**) module. AUC, area under the curve

Highly interacted genes in a module play a pivotal role in biological processes. Therefore, we selected the top 50 genes with the greatest biological relevance in the brown, blue, and red modules as hub genes. Furthermore, the PPI network of genes in each of the three modules was established in accordance with the STRING database. In the brown, red, and blue modules, there were 40, 29, and 28 overlapping hub genes, respectively, in both WGCNA and PPI analyses, which were selected as key genes. We screened 4141 DEGs between metastatic and non-metastatic OS samples using the limma package (adjusted P-value < 0.05, and |log2 (fold change)|> 0.2). The volcano plot of the DEGs is shown in Fig. 4d. As shown in Fig. 4a–c, the genes in the blue module does not show a closely interactive network; thus, the blue module was excluded from further analysis (Fig. 4b). We then overlapped the DEGs and hub genes in the brown and red modules using a Venn diagram. As shown in Fig. 4e, 40 genes are present in both DEGs and the brown module, and 25 genes are present in both DEGs and the red module. These 65 genes were considered key genes relevant to OS metastasis and were therefore selected for further analysis (Fig. 4e).

### Key genes play a prediction role in OS metastasis

To confirm the application of key genes in OS metastasis prediction, we chose the SVM model to classify the data set of metastatic and non-metastatic samples. We used the top 75% samples ranked randomly in GSE33382 and GSE21257 as the training set, and the remaining 25% samples as the test set. The sensitivity, specificity, and AUC of the key genes in the test set were obtained (Table 1, Fig. 4f–g). Gene list of ROC curves in brown module (Additional file 1: Table S1) and red module (Additional file 2: Table S2) were also provided as additional files. These results suggest that these genes can be potential biomarkers for OS metastasis prediction, and that this model is capable of discriminating patients with or without OS metastasis.

**Table 1:** The accuracy, sensitivity, specificity, PPV, NPV and AUC of SVM analysis of brown and red modules

Modules	Num of genes	Correct rate	Se	Sp	PPV	NPV	AUC
Brown							
1–5 genes	5	0.692	0.882	0.333	0.714	0.600	0.810
1–10 genes	10	0.731	0.938	0.400	0.714	0.800	0.743
1–15 genes	15	0.808	0.944	0.500	0.810	0.800	0.800
1–20 genes	20	0.808	0.900	0.500	0.857	0.600	0.733
1–25 genes	25	0.846	0.905	0.600	0.905	0.600	0.914
1–30 genes	30	0.885	1.000	0.450	0.857	1.000	0.933
1–35 genes	35	0.885	0.950	0.667	0.905	0.800	0.895
1–40 genes	40	0.923	1.000	0.714	0.905	1.000	0.905
Red							
1–5 genes	5	0.654	0.833	0.750	0.714	0.400	0.686
1–10 genes	10	0.808	0.900	0.500	0.857	0.600	0.743
1–15 genes	15	0.885	0.875	1.000	1.000	0.400	0.943
1–20 genes	20	0.885	0.910	0.750	0.952	0.600	0.857
1–25 genes	25	0.885	0.950	0.667	0.905	0.800	0.800

Se, sensitivity; Sp, specificity; PPV, positive prediction value; NPV, negative prediction value; AUC, area under ROC curve

### Identification of featured genes with TCGA dataset

**Fig. 5:**
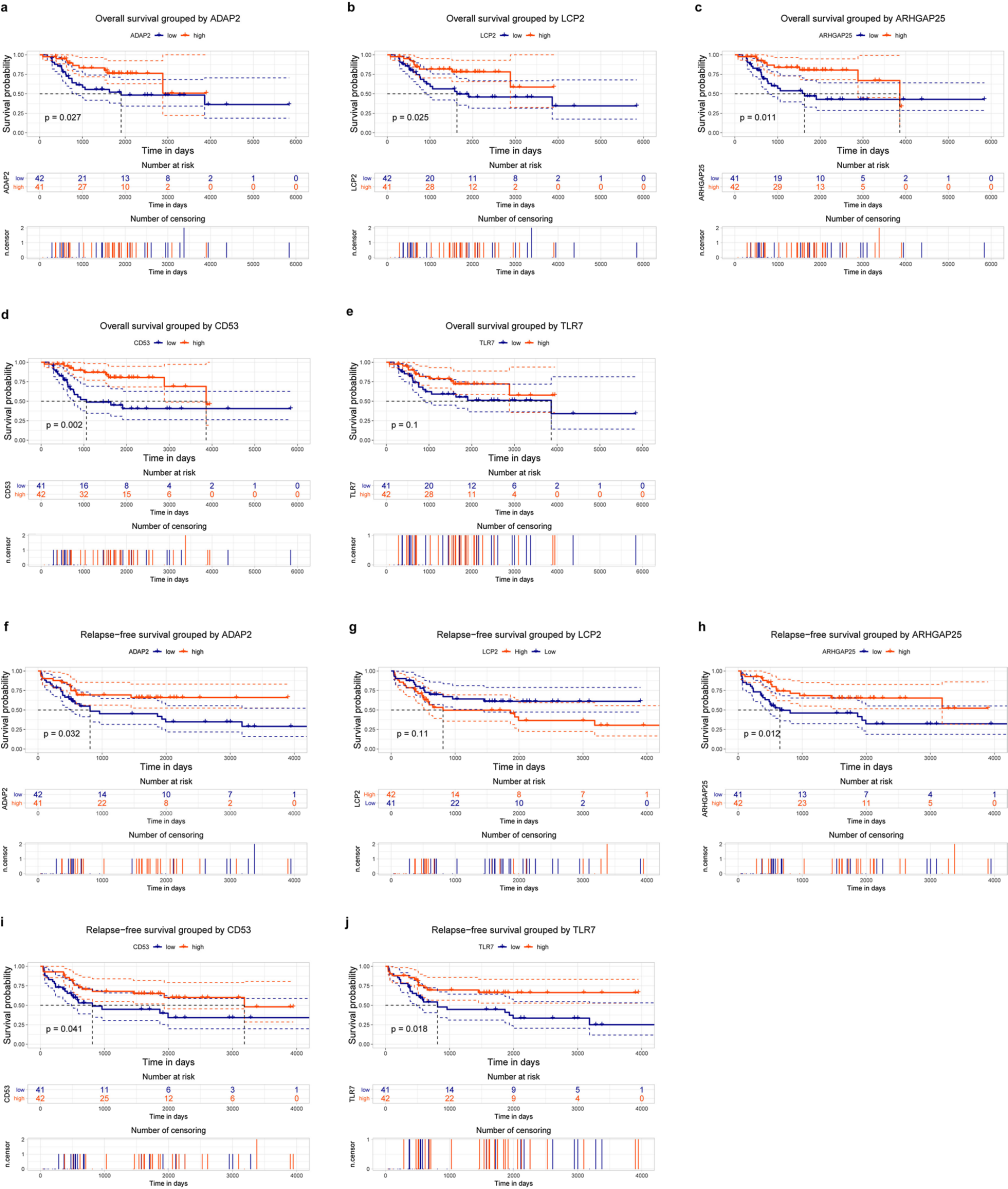
Key genes validation. ADAP2 (**a**), LCP2 (**b**), ARHGAP25 (**c**), CD53 (**d**) and TLR7 (**e**) expression levels were all downregulated in the metastatic OS compared with no-metastatic OS groups according to the Cancer Genome Atlas (TCGA) database

**Fig. 6:**
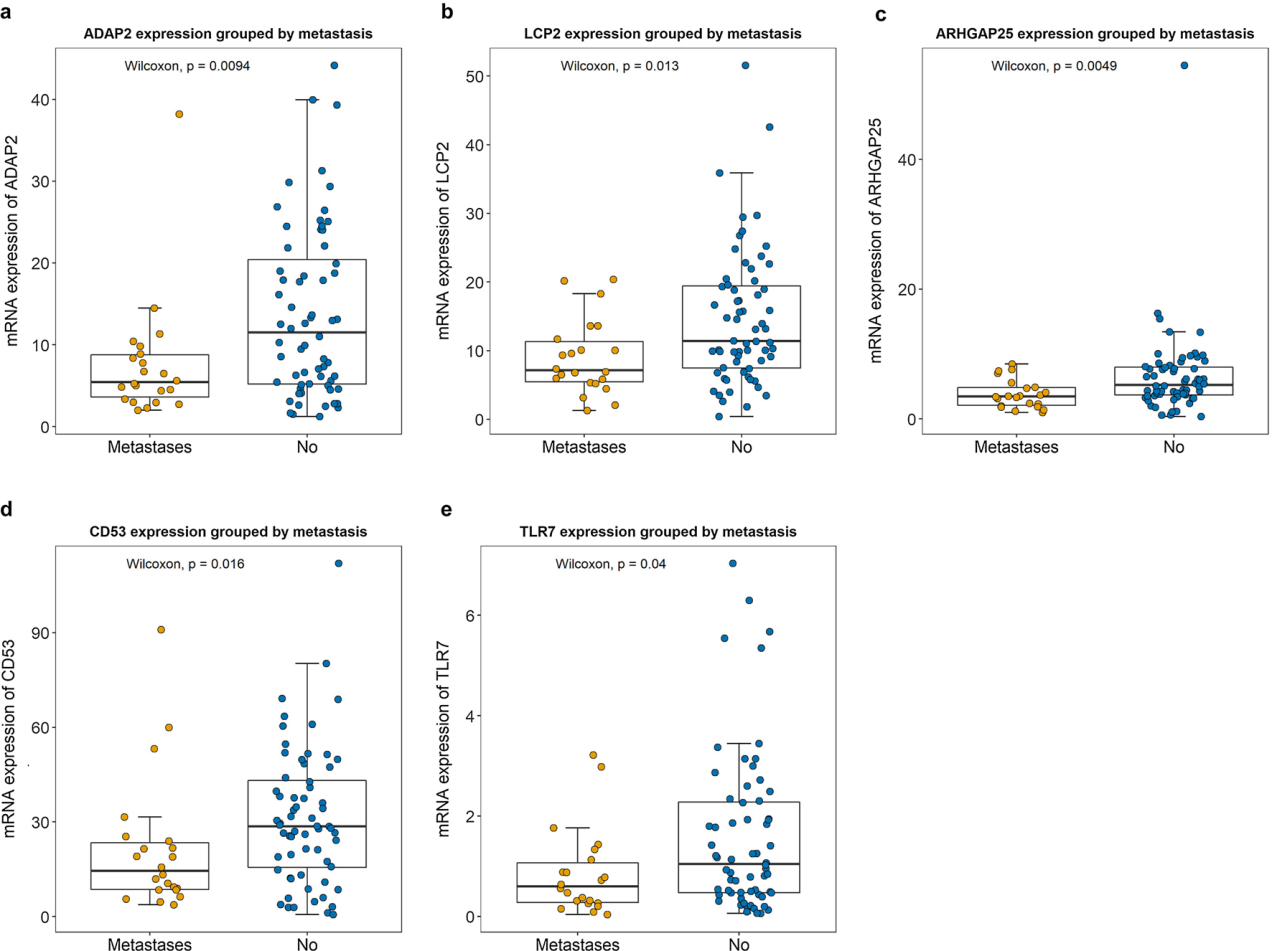
Survival analysis of association between ADAP2, LCP2, ARHGAP25, CD53 and TLR7 expression levels and survival rates in OS. **a**–**e** Overall survival analysis with ADAP2, LCP2, ARHGAP25, CD53 and TLR7 expression levels. **f**–**j** Relapse-free survival analysis with ADAP2, LCP2, ARHGAP25, CD53 and TLR7 expression levels

To evaluate the prognostic effect of the key genes in OS patients, the relationship between gene expression and survival time was determined using Kaplan–Meier survival analysis with the log-rank test. Finally, 17 key genes were found to be significantly correlated with survival time (overall survival time or relapse-free survival time). The expression analysis of the 17 key genes in TCGA dataset provides a unique insight into their function in OS metastasis. We measured the differences in the expression of the 17 key genes between metastatic and non-metastatic OS samples. As shown in Fig. 5, we found five genes with significantly lower expression in metastatic samples, namely ADAP2 (P = 0.0094), LCP2 (P = 0.013), ARHGAP25 (P = 0.0049), CD53 (P = 0.016), and toll-like receptor 7 (TLR7; P = 0.04). The association of these five genes with overall survival time and relapse-free survival time is shown in Fig. 6. The association of the remaining 12 key genes with survival time is shown in Additional file 4: Fig. S2.

### ARHGAP25 inhibited MG63 cell growth, migration, and epithelial–mesenchymal transition (EMT) progression in vitro

**Fig. 7:**
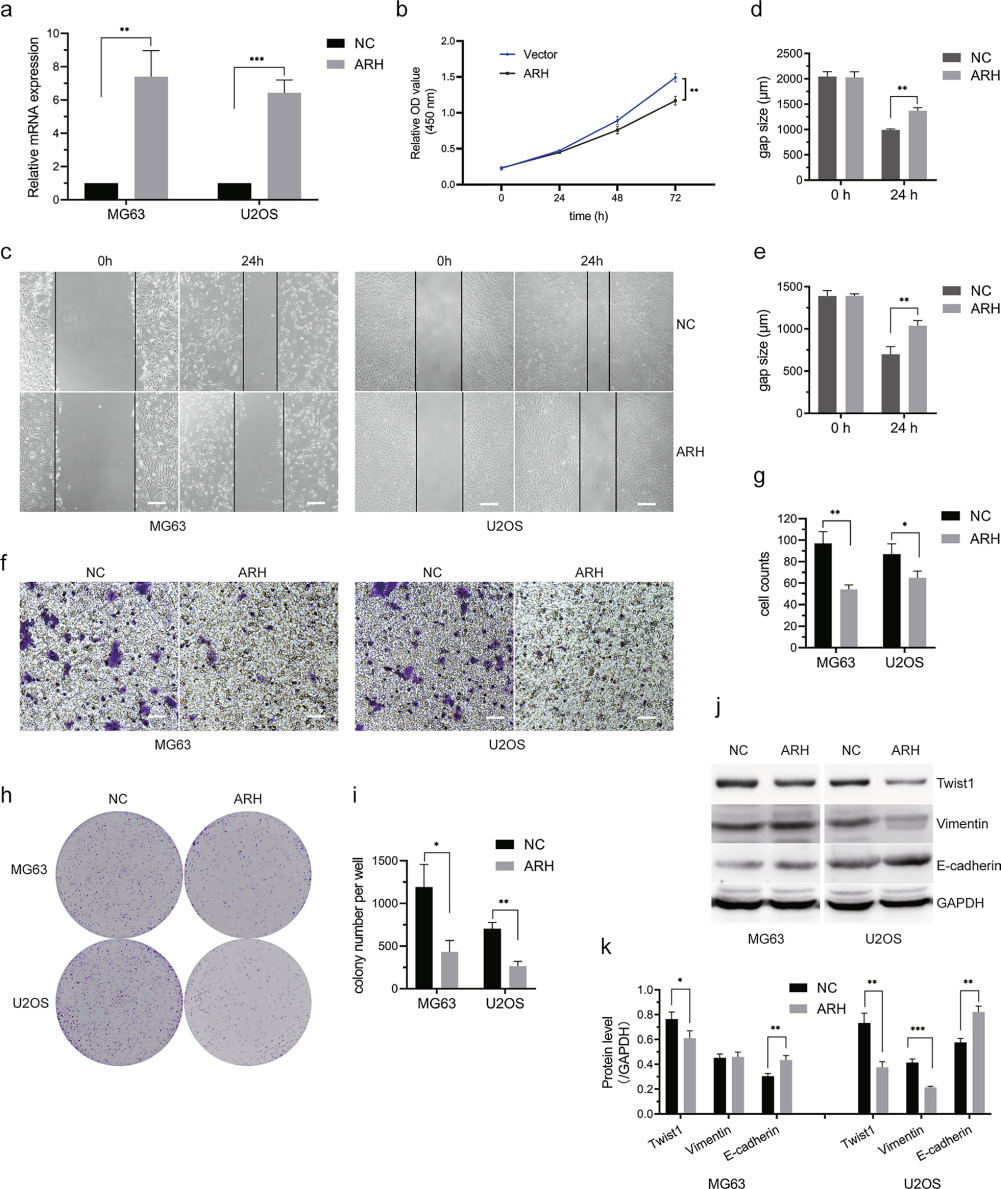
ARHGAP25 inhibited MG63 and U2OS cells’ growth, migration and invasion. **a** ARHGAP25 expression was determined in vector transfected MG63 and U2OS cells. **b** Overexpression of ARHGAP25 in MG63 and U2OS cells inhibited cell growth. **c** Representative images of MG63 and U2OS cells wound healing assay at 0 and 24 h after scratching. Bars indicate: 100 µm. **d**, **e** Quantification of the gap size in wound healing assay (n = 3), which showed that overexpression of ARHGAP25 suppressed wound healing. **f** Representative images of MG63 and U2OS cells stained with crystal violet in the transwell assay. Bars indicate: 100 µm. **g** Counts of cells invaded through the chambers (n = 3). **h** Representative images of colony formation assay to analyze cell proliferation in ARHGAP25 overexpressed OS cells. **i** Quantification of the colony number (n = 3). **j** Protein expression of Twist1, Vimentin, and E-cadherin was measured in OS cells with ARHGAP25 overexpression. **k** Quantification of protein expression using ImageJ. *Indicates P < 0.05, **indicates P < 0.01 and ***indicates P < 0.001. Picture **c**, **f** are magnified 100 times and h is not magnified

ARHGAP25 was selected to verify whether this integrated bioinformatics analysis works, as it was identified with a highly significant difference in all of the bioinformatic analyses. Targeting ARHGAP25 expression has been shown to inhibit the growth and migration of other cancer cells [19–21]. We employed CCK8 to examine relative cell growth and found that ARHGAP25 overexpression (Fig. 7a) in MG63 and U2OS cells was significantly lower than that in NC cells (Fig. 7b). The wound healing assay further suggested that MG63 and U2OS cells overexpressing ARHGAP25 had reduced migration-related abilities (Fig. 7c–e). To evaluate the effect of ARHGAP25 overexpression on OS cell invasion, we performed a transwell cell invasion assay. As shown in Fig. 7f–g, ARHGAP25 significantly inhibits MG63 and U2OS cell invasion. Moreover, the colony formation assay further confirmed that ARHGAP25 overexpression inhibited MG63 and U2OS cell growth (Fig. 7h–i). At the molecular level, western blotting results revealed that ARHGAP25 overexpression increased the expression of E-cadherin, an EMT-associated protein, and decreased the expression of EMT proteins Twist1 in MG63 and U2OS cells, and decreased vimentin expression in U2OS but not MG63 cells (Fig. 7j, k). The above data indicates that targeting ARHGAP25 expression suppresses OS cell growth, migration, and EMT progression.

## Discussion

OS is the most common bone cancer in children and adolescents and is characterized by a high propensity for metastasis [1], however, knowledge of the mechanism of OS metastasis is limited. Therefore, it is necessary to identify sufficient gene signatures with considerable efficacy in predicting metastasis status, which may improve the early diagnosis and clinical prognosis of OS. In this study, we employed WGCNA to establish a co-expression network and identified three modules that were most significantly associated with OS metastasis. By overlapping hub genes in the PPI network, corPvalueStudent analysis, and DEGs, we found that 40 and 25 key genes in brown and red modules, respectively, were significantly associated with OS metastasis. Using the SVM classifier, we precisely distinguished metastatic OS samples from non-metastatic samples using these key genes. However, none of the genes in the red module were significant in the overall or relapse-free survival analyses. TCGA database was used to further validate the prediction value of key genes in brown modules. Through a series of analyses of the clinical phenotypes and prognosis of OS, we established that five featured genes (ADAP2, LCP2, ARHGAP25, CD53, and TLR7) were significantly associated with OS metastasis and survival time.

ArfGAP with dual pleckstrin homology (PH) domain 2 (ADAP2), known as Centaurin-a2, is characterized by a C4-type zinc finger and two PH domains [22]. ADAP2 acts as a microtubule-associated protein that increases microtubule stability by interacting with β-tubulin. Microtubule stability ensures and maintains cell structure and function, and is critical in important cellular processes, including cell movement, division, and vesicular transport [23]. ADAP2 downregulation has been reported to promote cervical cancer HeLa cell proliferation [24]. Thus, a decrease in ADAP2 expression may play an important role in the cell division cycle and actin cytoskeleton to regulate OS cell proliferation and metastasis.

The SH2 domain-containing leukocyte protein of 76 kDa (SLP76, also known as LCP2) is expressed in most hematopoietic lineages. LCP2 plays a critical role in T-cell development and activation [25]. It is well known that T cells mediate anti-tumor activity in numerous tumors [26]. To date, there have been bioinformatics studies that showed that LCP2 may play a role in tumor occurrence and metastasis, such as colon cancer [27, 28] and glioblastoma multiforme [29]. To our knowledge, this is the first study on the association of LCP2 with OS metastasis, however, the mechanism requires further investigation.

CD53 is a member of the tetraspanin family, which is a group of cell surface proteins that participate in cell adhesion, motility, signal transduction, immune cell activation, tumor growth, and metastasis [30]. CD53 network genes were found to be poorly expressed in the high-metastasis breast cancer transplantation model and predicted distant metastasis-free survival specifically in ER+ breast cancers [31]. It has also been reported that CD53 is linked to tumor necrosis factor-α (TNF-α) expression by genome-wide association analysis [32], which may change the tumor immunogenic microenvironment and then affect the immune response [33]. In this study, we found that the CD53 mRNA expression level was lower in metastatic OS samples, which is in accordance with the decreased CD53 expression in high-metastasis breast cancer in a previous study [31]. Our results suggest a potential role of CD53-mediated immune response in OS metastasis.

TLR7 is a member of the TLR family that acts as a pattern recognition receptor and is expressed on the membrane of endosomes. TLR7 activation by ssRNA of virals and nucleic acids can induce the expression of type I interferon and inflammatory cytokines, and activate other immune cells through several signaling pathways, such as the NF-κB signaling pathway [34, 35]. Considering the powerful immune regulatory function of TLR7, the agonists of TLR7 have been approved for topical application in cancer treatment [36, 37]. Several studies have reported that the agonists of TLR7 could prevent tumor recurrence and eliminate metastasis [38, 39]. A previous bioinformatics analysis study reported that TLR7 signaling is related to OS metastasis [40]. This suggests that the TLR7 signaling pathway may be a potential target for OS metastasis therapy.

The Rac GTPase-activating protein 25 (ARHGAP25) has been confirmed to act as a negative regulator of several tumor metastases. In colorectal cancer, ARHGAP25 inhibits tumor metastasis via the Wnt/β-catenin pathway [41, 42]. A putative molecular network study showed that ARHGAP25 and LCP2 targeted more than five DEGs to play more important roles in colon cancer metastasis [27]. The RhoE/ROCK/ARHGAP25 pathway was reported to control alveolar rhabdomyosarcoma cell invasion [20]. Wnt signaling has been shown to promote tumor growth and metastasis in OS [5]. Considering that ARHGAP25 could inhibit the Wnt pathway to limit colorectal cancer, we assumed that ARHGAP25 could also limit OS metastasis, therefore, resulting in a reasonable decrease of ARHGAP25 expression in OS metastasis.

## Conclusions

We identified a five-gene signature as a practical and candidate biomarker for OS metastasis prediction based on data mining and analysis. In vitro validation demonstrated that ARHGAP25 overexpression reduced MG63 cell growth, migration, and invasion, indicating that ARHGAP25 can serve as a promising specific and prognostic biomarker for OS metastasis. Our results provide insights into the potential mechanisms of OS metastasis and candidate genes for the prediction of OS metastasis.

## Supplementary Information

**Additional file 1: Table S1.** Gene list of ROC curves in brown module

**Additional file 2: Table S2.** Gene list of ROC curves in red module

**Additional file 3: Figure S1. **Clustering of samples. Clustering was based on the expression data of GSE33382 and GSE21257.

**Additional file 4: Figure S2.** Survival analysis of association between the key genes expression levels except ADAP2, LCP2, ARHGAP25, CD53 and TLR7 and survival rates in OS.

## Data Availability

The datasets used and/or analyzed during the current study are available from the public database (https://www.ncbi.nlm.nih.gov/geo/; https://ocg.cancer.gov/programs/target/data-matrix) and the corresponding author on reasonable request.

## Abbreviations

OS: Osteosarcoma
WGCNA: Weighted gene co-expression network analysis
SVM: Support vector machine
GO: Gene ontology
KEGG: Kyoto Encyclopedia of Genes and Genomes
ROC: Receiver operating characteristic
TCGA: The Cancer Genome Atlas
PPI: Protein–protein interaction
GEO: Gene Expression Omnibus
FPKM: Fragments per million
MEs: Module eigengenes
Se: Sensitivity
Sp: Specificity
AUC: Area under the curve
PPV: Positive predictive value
NPV: Negative predictive value
ARHGAP25: Rac GTPase-activating protein 25()
TLR7: Toll like receptor 7
