# Supplementary material for: Identification of key genes as predictive biomarkers for osteosarcoma metastasis using translational bioinformatics
Source: Cancer Cell Int. 2021 Dec 2;21:640. doi: 10.1186/s12935-021-02308-w (PMC8638136; doi:10.1186/s12935-021-02308-w)
Supplement: Supplementary file 1 — Additional file 1: Table S1. Gene list of ROC curves in brown module [file 12935_2021_2308_MOESM1_ESM.doc]

Table S1. Gene list of ROC curves in brown module

| Ensembl_gene_ID | Symbol | CytoHubba score | WGCNA score | AveExpr | P.Value |  |  |  |  |  |  |  |  |
| --- | --- | --- | --- | --- | --- | --- | --- | --- | --- | --- | --- | --- | --- |
| ENSG00000186517 | ARHGAP30 | 10 | 0.93665 | 3.141732 | 4.87112E-08 | 5 genes AUC 0.81 | 10 genes AUC 0.743 | 15 genes AUC 0.8 | 20 genes AUC 0.733 | 25 genes AUC 0.914 | 30 genes AUC 0.933 | 35 genes AUC 0.895 | 40 genes AUC 0.905 |
| ENSG00000140968 | IRF8 | 10 | 0.936592 | 3.181612 | 2.50597E-08 |
| ENSG00000110324 | IL10RA | 10 | 0.930175 | 3.109209 | 3.61013E-07 |
| ENSG00000143119 | CD53 | 10 | 0.926926 | 3.12476 | 7.85888E-07 |
| ENSG00000134516 | DOCK2 | 10 | 0.921397 | 3.236337 | 8.51588E-08 |
| ENSG00000187474 | FPR3 | 10 | 0.894843 | 3.243917 | 4.65326E-09 |  |
| ENSG00000137491 | SLCO2B1 | 10 | 0.870815 | 3.25203 | 4.92759E-08 |  |
| ENSG00000141968 | VAV1 | 9 | 0.944329 | 2.989304 | 1.73192E-06 |  |
| ENSG00000101336 | HCK | 9 | 0.933872 | 3.109771 | 2.23519E-06 |  |
| ENSG00000100060 | MFNG | 9 | 0.927048 | 3.136881 | 8.73552E-07 |  |
| ENSG00000043462 | LCP2 | 9 | 0.920987 | 3.271961 | 7.49334E-06 |  |  |
| ENSG00000111679 | PTPN6 | 9 | 0.915696 | 2.875507 | 0.020267299 |  |  |
| ENSG00000163131 | CTSS | 9 | 0.911553 | 3.059319 | 2.40715E-08 |  |  |
| ENSG00000127951 | FGL2 | 9 | 0.910859 | 3.200293 | 3.02838E-10 |  |  |
| ENSG00000012779 | ALOX5 | 9 | 0.910293 | 3.245967 | 1.66237E-07 |  |  |
| ENSG00000103490 | PYCARD | 9 | 0.904501 | 3.302738 | 4.53208E-07 |  |  |  |
| ENSG00000112799 | LY86 | 9 | 0.902397 | 3.225977 | 1.81721E-07 |  |  |  |
| ENSG00000142347 | MYO1F | 9 | 0.88888 | 2.967707 | 6.34947E-05 |  |  |  |
| ENSG00000184060 | ADAP2 | 9 | 0.885596 | 3.29427 | 1.01968E-06 |  |  |  |
| ENSG00000175463 | TBC1D10C | 9 | 0.882744 | 3.005941 | 5.31402E-06 |  |  |  |
| ENSG00000167261 | DPEP2 | 9 | 0.874999 | 2.98286 | 7.67673E-07 |  |  |  |  |
| ENSG00000155659 | VSIG4 | 9 | 0.87326 | 3.192199 | 8.90434E-07 |  |  |  |  |
| ENSG00000110934 | BIN2 | 8 | 0.951765 | 3.020633 | 2.45048E-07 |  |  |  |  |
| ENSG00000102879 | CORO1A | 8 | 0.931453 | 3.160237 | 2.03824E-06 |  |  |  |  |
| ENSG00000158714 | SLAMF8 | 8 | 0.907491 | 3.049858 | 2.33768E-06 |  |  |  |  |
| ENSG00000171631 | P2RY6 | 8 | 0.890269 | 2.900054 | 0.000906052 |  |  |  |  |  |
| ENSG00000175489 | LRRC25 | 8 | 0.884959 | 2.976983 | 9.12268E-08 |  |  |  |  |  |
| ENSG00000198771 | RCSD1 | 8 | 0.883993 | 2.972342 | 0.000151207 |  |  |  |  |  |
| ENSG00000115956 | PLEK | 8 | 0.879488 | 3.293779 | 1.34645E-05 |  |  |  |  |  |
| ENSG00000122122 | SASH3 | 7 | 0.953762 | 3.035408 | 2.24967E-06 |  |  |  |  |  |
| ENSG00000104894 | CD37 | 7 | 0.931339 | 3.080659 | 1.0032E-08 |  |  |  |  |  |  |
| ENSG00000117091 | CD48 | 7 | 0.912913 | 3.119981 | 1.47084E-06 |  |  |  |  |  |  |
| ENSG00000126264 | HCST | 7 | 0.912387 | 3.362319 | 5.19519E-08 |  |  |  |  |  |  |
| ENSG00000163219 | ARHGAP25 | 7 | 0.900434 | 3.075476 | 3.09904E-06 |  |  |  |  |  |  |
| ENSG00000102524 | TNFSF13B | 7 | 0.896687 | 3.123221 | 3.05467E-06 |  |  |  |  |  |  |
| ENSG00000196664 | TLR7 | 7 | 0.869276 | 3.042187 | 2.0292E-08 |  |  |  |  |  |  |  |
| ENSG00000136250 | AOAH | 6 | 0.885384 | 3.013457 | 8.69203E-07 |  |  |  |  |  |  |  |
| ENSG00000174004 | NRROS | 6 | 0.87697 | 3.046278 | 4.56621E-05 |  |  |  |  |  |  |  |
| ENSG00000005844 | ITGAL | 6 | 0.87045 | 2.949594 | 0.000287635 |  |  |  |  |  |  |  |
| ENSG00000152315 | KCNK13 | 6 | 0.865709 | 2.947977 | 2.84957E-07 |  |  |  |  |  |  |  |
