# Supplementary material for: Identification of key genes as predictive biomarkers for osteosarcoma metastasis using translational bioinformatics
Source: Cancer Cell Int. 2021 Dec 2;21:640. doi: 10.1186/s12935-021-02308-w (PMC8638136; doi:10.1186/s12935-021-02308-w)
Supplement: Supplementary file 2 — Additional file 2: Table S2. Gene list of ROC curves in red module [file 12935_2021_2308_MOESM2_ESM.doc]

Table S2. Gene list of ROC curves in red module

| Ensembl_gene_ID | Symbol | CytoHubba score | WGCNA score | AveExpr | P.Value |  |  |  |  |  |
| --- | --- | --- | --- | --- | --- | --- | --- | --- | --- | --- |
| ENSG00000127423 | AUNIP | 11 | 0.871356786 | 3.017345044 | 0.000257289 | 5 genes AUC 0.686 | 10 genes AUC 0.743 | 15 genes AUC 0.943 | 20 genes AUC 0.857 | 25 genes AUC 0.8 |
| ENSG00000165490 | DDIAS | 11 | 0.845061884 | 3.089154155 | 0.01792776 |
| ENSG00000146670 | CDCA5 | 10 | 0.892718008 | 3.285476779 | 9.42482E-05 |
| ENSG00000156970 | BUB1B | 10 | 0.882613855 | 3.148148824 | 0.000251515 |
| ENSG00000154839 | SKA1 | 10 | 0.853092292 | 3.021837441 | 0.017231299 |
| ENSG00000197299 | BLM | 9 | 0.880253175 | 3.017115292 | 0.02562418 |  |
| ENSG00000134222 | PSRC1 | 9 | 0.859493964 | 3.14102841 | 0.011953391 |  |
| ENSG00000145386 | CCNA2 | 9 | 0.858152232 | 3.22458844 | 0.000674463 |  |
| ENSG00000075218 | GTSE1 | 9 | 0.849844523 | 3.107672995 | 0.003303933 |  |
| ENSG00000051341 | POLQ | 9 | 0.825705345 | 3.05551478 | 0.012059599 |  |
| ENSG00000111206 | FOXM1 | 9 | 0.816034831 | 3.083567284 | 0.000276589 |  |  |
| ENSG00000117399 | CDC20 | 9 | 0.800395092 | 3.510395518 | 0.011365965 |  |  |
| ENSG00000115163 | CENPA | 8 | 0.876390146 | 3.172557852 | 0.023232007 |  |  |
| ENSG00000142731 | PLK4 | 8 | 0.868549525 | 3.165390464 | 0.020644302 |  |  |
| ENSG00000101412 | E2F1 | 8 | 0.860731803 | 3.006823714 | 0.047193497 |  |  |
| ENSG00000093009 | CDC45 | 8 | 0.853058148 | 3.017686744 | 0.002059688 |  |  |  |
| ENSG00000198901 | PRC1 | 8 | 0.845255155 | 3.383449361 | 0.036298837 |  |  |  |
| ENSG00000149554 | CHEK1 | 8 | 0.844674315 | 3.090402714 | 0.004288525 |  |  |  |
| ENSG00000117724 | CENPF | 8 | 0.794346594 | 3.215143973 | 0.011010312 |  |  |  |
| ENSG00000135476 | ESPL1 | 7 | 0.821375744 | 3.004999334 | 0.022217319 |  |  |  |
| ENSG00000152253 | SPC25 | 7 | 0.813040256 | 2.986261137 | 6.79039E-05 |  |  |  |  |
| ENSG00000090889 | KIF4A | 6 | 0.830973432 | 3.111518815 | 0.015164031 |  |  |  |  |
| ENSG00000065328 | MCM10 | 5 | 0.852144727 | 3.025803102 | 2.33971E-05 |  |  |  |  |
| ENSG00000163808 | KIF15 | 5 | 0.832889079 | 3.032470246 | 0.001718197 |  |  |  |  |
| ENSG00000087586 | AURKA | 5 | 0.814726842 | 3.301365701 | 0.04638823 |  |  |  |  |
