# Supplementary figures and images for: Identification of key genes as predictive biomarkers for osteosarcoma metastasis using translational bioinformatics
Source: Cancer Cell Int. 2021 Dec 2;21:640. doi: 10.1186/s12935-021-02308-w (PMC8638136; doi:10.1186/s12935-021-02308-w)

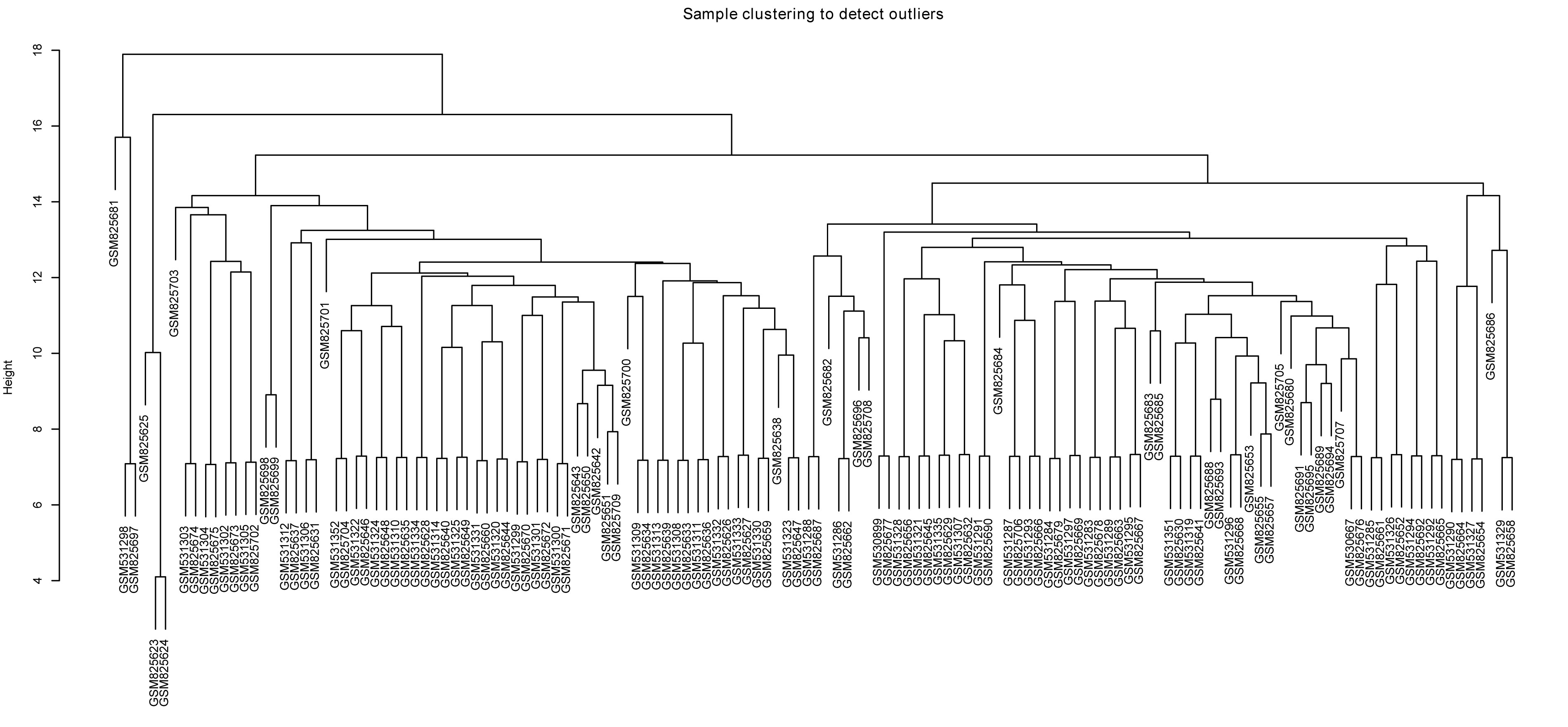

Supplement: Supplementary file 3 — Additional file 3: Figure S1. Clustering of samples. Clustering was based on the expression data of GSE33382 and GSE21257. [file 12935_2021_2308_MOESM3_ESM.jpg]

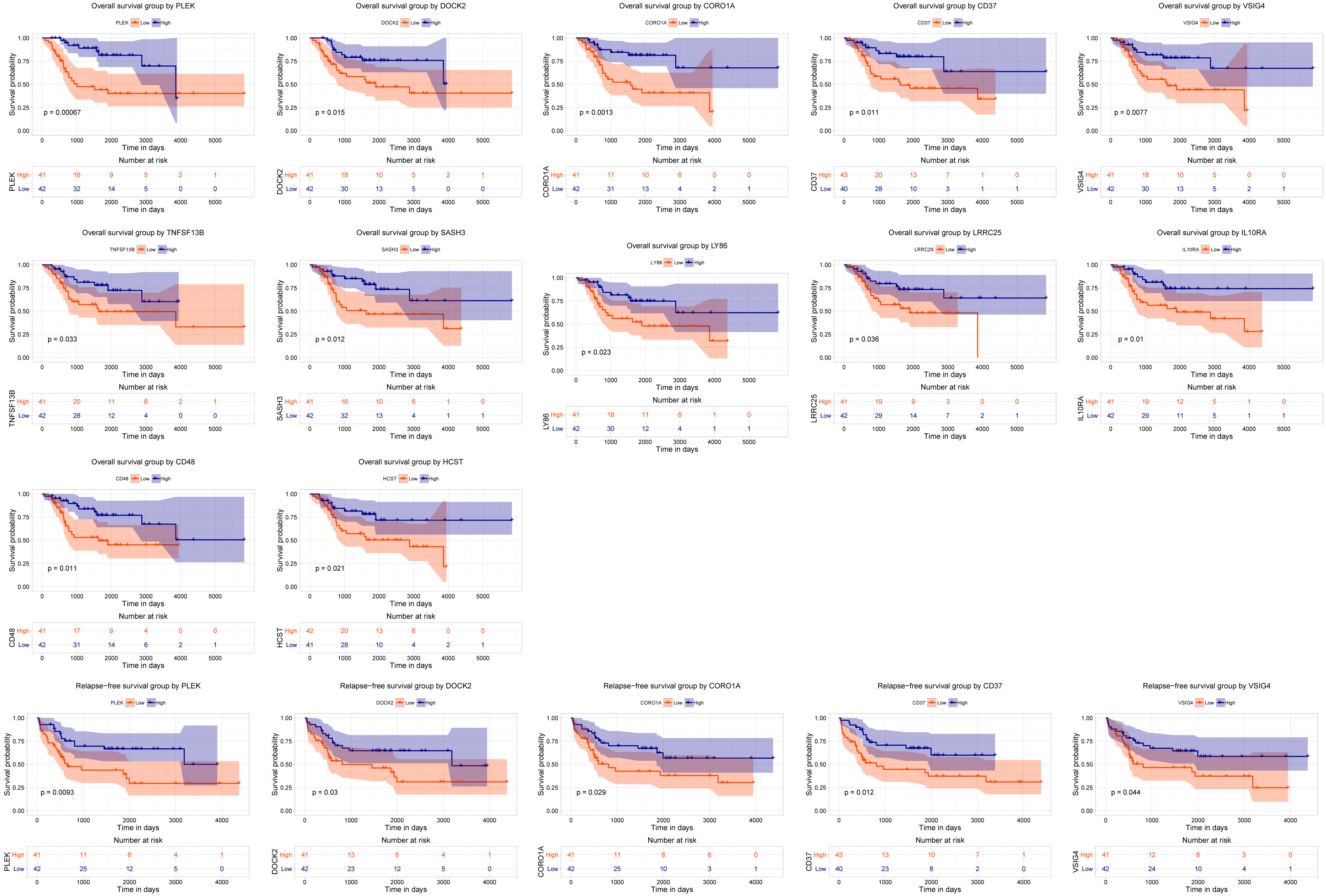

Supplement: Supplementary file 4 — Additional file 4: Figure S2. Survival analysis of association between the key genes expression levels except ADAP2, LCP2, ARHGAP25, CD53 and TLR7 and survival rates in OS. [file 12935_2021_2308_MOESM4_ESM.jpg]
